# Direct comparison of different therapeutic cell types susceptibility to inflammatory cytokines associated with COVID-19 acute lung injury

**DOI:** 10.1186/s13287-021-02699-7

**Published:** 2022-01-15

**Authors:** Ramana Vaka, Saad Khan, Bin Ye, Yousef Risha, Sandrine Parent, David Courtman, Duncan J. Stewart, Darryl R. Davis

**Affiliations:** 1 University of Ottawa Heart Institute, Division of Cardiology, Department of Medicine, University of Ottawa, H3214, 40 Ruskin Street, Ottawa, ON K1Y 4W7 Canada; 2 Department of Cellular and Molecular Medicine, University of Ottawa, Ottawa, K1H8M5 Canada; 3 Ottawa Hospital Research Institute, Division of Regenerative Medicine, Department of Medicine, University of Ottawa, Ottawa, K1H8L6 Canada

**Keywords:** Stem cells, COVID-19, Acute respiratory distress syndrome, Cytokines, Extracellular vesicles

## Abstract

**Background:**

Although 90% of infections with the novel coronavirus 2 (COVID-19) are mild, many patients progress to acute respiratory distress syndrome (ARDS) which carries a high risk of mortality. Given that this dysregulated immune response plays a key role in the pathology of COVID-19, several clinical trials are underway to evaluate the effect of immunomodulatory cell therapy on disease progression. However, little is known about the effect of ARDS associated pro-inflammatory mediators on transplanted stem cell function and survival, and any deleterious effects could undermine therapeutic efficacy. As such, we assessed the impact of inflammatory cytokines on the viability, and paracrine profile (extracellular vesicles) of bone marrow-derived mesenchymal stromal cells, heart-derived cells, and umbilical cord-derived mesenchymal stromal cells.

**Methods:**

All cell products were manufactured and characterized to established clinical release standards by an accredited clinical cell manufacturing facility. Cytokines and Extracellular vesicles in the cell conditioned media were profiled using proteomic array and nanoparticle tracking analysis. Using a survey of the clinical literature, 6 cytotoxic cytokines implicated in the progression of COVID-19 ARDS. Flow cytometry was employed to determine receptor expression of these 6 cytokines in three cell products. Based on clinical survey and flow cytometry data, a cytokine cocktail that mimics cytokine storm seen in COVID-19 ARDS patients was designed and the impact on cytokine cocktail on viability and paracrine secretory ability of cell products were assessed using cell viability and nanoparticle tracking analysis.

**Results:**

Flow cytometry revealed the presence of receptors for all cytokines but IL-6, which was subsequently excluded from further experimentation. Despite this widespread expression, exposure of each cell type to individual cytokines at doses tenfold greater than observed clinically or in combination at doses associated with severe ARDS did not alter cell viability or extracellular vesicle character/production in any of the 3 cell products.

**Conclusions:**

The paracrine production and viability of the three leading cell products under clinical evaluation for the treatment of severe COVID-19 ARDS are not altered by inflammatory mediators implicated in disease progression.

**Supplementary Information:**

The online version contains supplementary material available at 10.1186/s13287-021-02699-7.

## Background

Although 90% of infections with the novel coronavirus 2 (SARS-CoV-2) are mild, a quarter of all hospitalized patients require admission to the intensive care unit [1]. Like many infectious agents, SARS-CoV-2 results in diffuse alveolar damage and a toxic cytokine storm mediated by a hyperactive innate immune response involving pro-inflammatory macrophages and granulocytes [2–4]. This hyperinflammatory state leads to diffuse alveolar and pulmonary interstitial inflammation which impairs oxygenation and triggers extensive immunothrombosis [5–8]. Clinically, this constellation of symptoms is referred to as acute respiratory distress syndrome (ARDS) and often requires mechanical ventilation which carries a 50–90% risk of death [1]. Given the key role of innate immunity in the pathogenesis of ARDS, immunomodulatory cell therapy has the potential to make a significant impact on COVID-19 outcomes.

The immunomodulatory benefits seen after mesenchymal stromal cell (MSC) treatment [9–11] have been reproduced by multiple groups around the world [12–14]. Recently, a number of cell therapy-based trials have been initiated world-wide for the treatment of COVID-19 patients in the intensive care setting (50 + trials registered on clinicaltrials.org, accessed September 14, 2021; Additional file 1: Table S1). Of these, preliminary results have shown promise [15–18] but there are no full data sets yet published that prove cell treatment will provide tangible benefits in COVID-19 ARDS.

Although cell therapy may offer a complimentary treatment for COVID-19 ARDS, there are several roadblocks to effective clinical translation. Firstly, the optimal cell product is not clear. In early 2020, the prospect of recurrent waves of critically ill COVID-19 patients requiring intensive care prompted the use of readily available cell sources, which included bone marrow mesenchymal stromal cells (BM-MSCs), heart-derived cells (HDCs), and umbilical cord derived stromal cells (UC-MSCs). As shown in Additional file 1: Table S1, these cell of diverse tissue origin (i.e., aged adult heart tissue from patient undergoing open chest surgery vs. umbilical cord tissue vs. young adult bone marrow) comprise most of the cell products currently under clinical investigation. To date, there is no data on the advantages or disadvantages of these choices which makes comparison of trial data challenging. Second, several of the pre-clinical papers focusing on cell therapies for ARDS rely on cell manufactured under non-GMP, research-grade cells. These cells often perform very differently from cell products cultured within clinical cell manufacturing facilities using established GMP-compatible protocols [19]. Third, the effect of the pro-inflammatory cytokines triggered by COVID-19 ARDS on transplanted cells has not been fully characterized. This cell-host interaction is important as many of the cytokines produced in response to COVID-19 (i.e., interleukin-1 beta (IL-1β), IL-2, IL-6, IL-8, IL-10, and tumor necrosis factor alpha (TNFα)) [20–23] stimulate infiltration of macrophages, monocytes, and neutrophils into the lung tissue. Further, pro-inflammatory cytokines can induce endothelial cell apoptosis which results in microvascular damage, alveolar edema, and hypoxia [24]. In the COVID-19 cell trials, all cell products will be injected into the venous system which delivers them into the inflamed hypoxic lungs. Cells then lodge in the lungs and are expected to survive while delivering a repertoire of salutary paracrine immunomodulatory signals that consist of cytokines and extracellular vesicles (EVs).

To address these issues, we compared different cell products that are cultured in a cell manufacturing facility using protocolized GMP-compatible standards for the treatment of COVID-19 ARDS patients. Although UC-MSCs are a generally easily accessible cell type, we were fortunate to have BM-MSC and HDC cell products on hand which prompted us to include all 3 cell products in the study. We designed a custom cytokine cocktail from the current clinical literature to simulate the effects of toxic COVID-19 cytokines on transplanted cell survival and activity. Given the importance of paracrine mechanisms for the therapeutic effects of MSC therapies, we then focused on the soluble substances and EVs produced by these cells. Comparing different cell products and understanding the impact of COVID-19 ARDS associated cytokines on transplanted cell survival and function, we hope to provide much needed insights into the results from clinical trials already underway.

## Methods

### Cell culture

BM-MSCs were isolated from bone marrow samples collected from young healthy volunteers, who were enrolled in the CISS-1 trial under a protocol approved by the Ottawa Hospital Research Ethics Board [25]. Cells were cultured to clinical grade standards in the cell manufacturing facility at the Ottawa Hospital Research Institute following established protocols [25]. The cells were cultured in GMP-grade MSC NutriStem® XF medium (SARTORIUS) under constant 21% oxygen conditions.

HDCs were cultured from left atrial appendages donated under a protocol approved by the University of Ottawa Heart Institute Research Ethics Board in the Ottawa Hospital Research Institute cell manufacturing facility, as previously described [26–28]. Briefly, atrial tissue was minced and digested with collagenase IV (Roche) before plating on fibronectin coated plates in GMP-grade MSC NutriStem® XF medium (SARTORIUS) under constant 5% oxygen conditions [26]. HDCs that spontaneously emerged as an outgrowth on the plate surface were harvested using TrypLE Select (Life Technologies) once a week prior to expansion under adherent conditions for 1 week before direct experimentation.

In collaboration with the Centre for Regenerative Therapies in Dresden, Germany, we isolated UC-MSCs from full GMP primary UC cell isolates under a protocol approved by the Ottawa Hospital Research Ethics Board [29]. Isolated UC-MSCs were cultured in high glucose Dulbecco's Modified Eagle Medium (DMEM) basal medium (ThermoFisher Scientific) with 10% clinical grade platelet lysate (PLTGold100 GMP, Mill Creek Life Sciences) under constant 5% oxygen conditions.

### Cell surface marker characterization

As per guidelines set by International Society for Cellular Therapy, MSCs need to express a series characteristic cell surface markers (i.e., CD73, CD90 and CD105) and not display markers indicative of hematopoietic cell identity (i.e., CD14, CD19, CD34, CD45 and HLA-DR) (30). Thus, we used flow cytometry to characterize these markers on both BM or UC-MSCs. Harvested BM-MSCs or UC-MSCs were washed with flow buffer (2% FBS in PBS) and stained with the following antibodies all conjugated to PE: CD73 (Biolegend, 344004), CD90 (555596BD, Biosciences), CD105 (MHCD10504, Invitrogen), D11b (A18675, Life Technologies), CD14 (55398, BD Biosciences), CD19 (555822, BD Biosciences), CD34 (555822, BD Biosciences), CD45 (555483, BD Biosciences), CD79a (561942, BD Biosciences), CD44 (550986, BD Biosciences), CD166 (559263, BD Biosciences) and HLA-DR (555813, BD Biosciences). The cells were then stained for 30 min at 4 °C and washed once with flow buffer prior to data collection on the Attune NXT Flow Cytometer (Life Technologies). The collected data were analyzed using Flow Jo V10.

### Indoleamine 2,3-dioxygenase gene expression

Indoleamine 2,3-dioxygenase (IDO) expression in the cell products was measured using quantitative reverse transcription polymerase chain reaction. Briefly, harvested cells were pelleted, and RNA was isolated (74104, Qiagen) and reverse transcribed (205311, Qiagen). The resultant cDNA was amplified using validated primers for IDO (Hs_IDO1_1_SG) and GAPDH (Hs_GAPDH_1_SG) with amplification quantification using SYBR Green (204143, Qiagen) on a BioRad CFX96.

### Tissue factor activity assay

Tissue factor (TF) activity in cell lysates was measured using a human Tissue Factor Activity Assay (108906, Abcam) according to the manufacturer’s directions. Briefly, harvested cells were pelleted and lysed with Octy-Beta-D-glycopyranoside (O9001, Sigma-Aldrich). Total protein was measured using a Bradford assay with a bovine serum albumin standard. Ten microliters of sample or Tissue Factor standard was loaded into each well and mixed with equal volumes of Factor VII and Factor X. The plate contents were mixed and incubated at 37 °C for 30 min in a humidified incubator. After incubation, the Factor Xa substrate was added, and absorbance was measured at 405 nm every 3 min for 45 min at 37 °C.

### Angiotensin converting enzyme gene expression

Angiotensin converting enzyme **(**ACE-2) expression in all 3 cell products was measured using a quantitative reverse transcription polymerase chain reaction. In brief, harvested cells were pelleted and RNA was isolated (74104, Qiagen). The isolated RNA was reverse transcribed (20531, Qiagen) and cDNA was amplified using primers for ACE-2 (Hs_ACE2_1_SG) and GAPDH (Hs_GAPDH_1_SG). Amplification was quantified using SYBR green (204143, Qiagen) on a BioRad CFX96.

### Cytokine and EV profiling

To generate conditioned media for experimentation and to reflect the hypoxic lung milieu, cells were grown in their respective basal media (BM-MSC/HDCs: Nutristem basal media; UC-MSC: DMEM alone) at 1% oxygen for 48 h. EVs were isolated using ultracentrifugation (10,000×g 30 min and 100,000×g 3 h [31, 32]. EV content, size, and surface marker expression were analyzed using nanoparticle tracking analysis (NanoSight LM10) and the Exo-Check Exosome Antibody Array (EXORAY200B, Systems Biosciences), respectively.

The cytokine content within conditioned media collected from a separate set of cultures and was evaluated using a proteomic array (ARY022B, RD Systems). Briefly, the nitrocellulose membranes with captured antibodies for 105 different human cytokines were incubated with cell culture supernatants overnight at 4 °C. The membranes were washed before the addition of detection antibody for 1 h and streptavidin-HRP for 30 min followed by scanning the membranes using film processor (SRX-101A, Konica Minolta). Pixel densities on the developed X-ray film were collected and analyzed using image-J analysis. Pixel density of negative control spots (background) in each sample was subtracted from the positive signal pixel densities of all the cytokines in the respective biological replicate. The detailed information on the array coordinates and cytokines analyzed are included in the Additional file 2: Fig. S1.

### Modeling toxic COVID-19 cytokines

To identify cytokines implicated in COVID-19 ARDS, we have performed a survey of clinical literature using 3 databases (search strategy is presented in Additional file 1: Table S2). Flow cytometry was then used to evaluate if transplanted cells expressed receptors for cytokines found in COVID-19 ARDS. BM-MSCs, HDCs and UC-MSCs were stained with anti-human IL-1 receptor 1 (IL-1R1, SC-393998FITC, Santa Cruz Biotechnology), IL-2 receptor (IL-2R, 339009, Biolegend), IL-6 receptor (IL-6R, 46-1269-42, ThermoFisher Scientific), IL-8 receptor (IL-8RA, 320622, Biolegend), IL-10 receptor (IL-10R, 308811, Biolegend), and TNF-α receptor 1 (TNFR1, MBS1800669, MyBiosource) antibodies at predetermined optimal dilutions. Appropriate isotype controls were used to correct compensation and to confirm antibody specificity. Cell data was acquired on a BD FACSAria IIu (BD Biosciences) and analyzed using FlowJo (V10.7.0).

Based on the cytokine receptor expression profile determined using flow cytometry, a dose–response effect of individual cytokines was performed using a colorimetric cell viability assay (CCK-8; Dojindo Molecular Technologies) to screen for direct toxic effects. Cytokines and dose response assay information are presented in Additional file 1: Table S4. All measurements were performed using a calibrated plate reader (BioTek Synergy Mx) with 3 technical replicates per biological sample. Cell viability data are presented after background subtraction on the absorbance signal measured at 450 nm with absorbance indicative of viable cells expressed as a percentage of the baseline (no cytokine treatment) group. Further, to assess the impact of ARDS associated cytokine storm on these cells, we measured cell viability and EV character and yield upon treatment with combination of cytokines. Given most intravenous delivered cells reside within the injured lungs for only 2–3 days [33], cells were exposed to individual cytokines or a cocktail of all relevant cytokines found in COVID-19 ARDS lungs for 48 h prior to end point measurements.

### Endothelial cell permeability assay

Human Pulmonary Microvascular Endothelial Cells (ScienCell Research Laboratories) were seeded on collagen (C3867, Millipore Sigma) coated transwell inserts (3413, Corning) [34, 35]. After 36 h, inserts were treated with LPS (100 µg/mL, L4391, Millipore Sigma) and control UC-MSC conditioned media or cytokine cocktail treated UC-MSC conditioned media. After 6 h, conditioned media was aspirated, and cell permeability was evaluated using fluorescence (BioTek Synergy Mx) for FITC-Dextran (46944, Millipore Sigma).

### Statistical analysis

All statistical tests and graphical depictions of data are defined within the figure legends for the data panels. All data are presented as individual and mean values ± standard error of mean. To determine if differences existed within groups, data were analyzed by student’s *t* test or a one-way or two-way analysis of variance (ANOVA; GraphPad Prism v. 9.1) with post hoc testing using Tukey’s or Dunnett’s Multiple Comparisons test to determine the group(s) with the difference(s). In all cases, variances were assumed to be equal and normality was confirmed prior to further post-hoc testing. A final value of *p* ≤ 0 + 0.05 was considered significant for all analyses.

## Results

### GMP cultured cell products

**Fig. 1:**
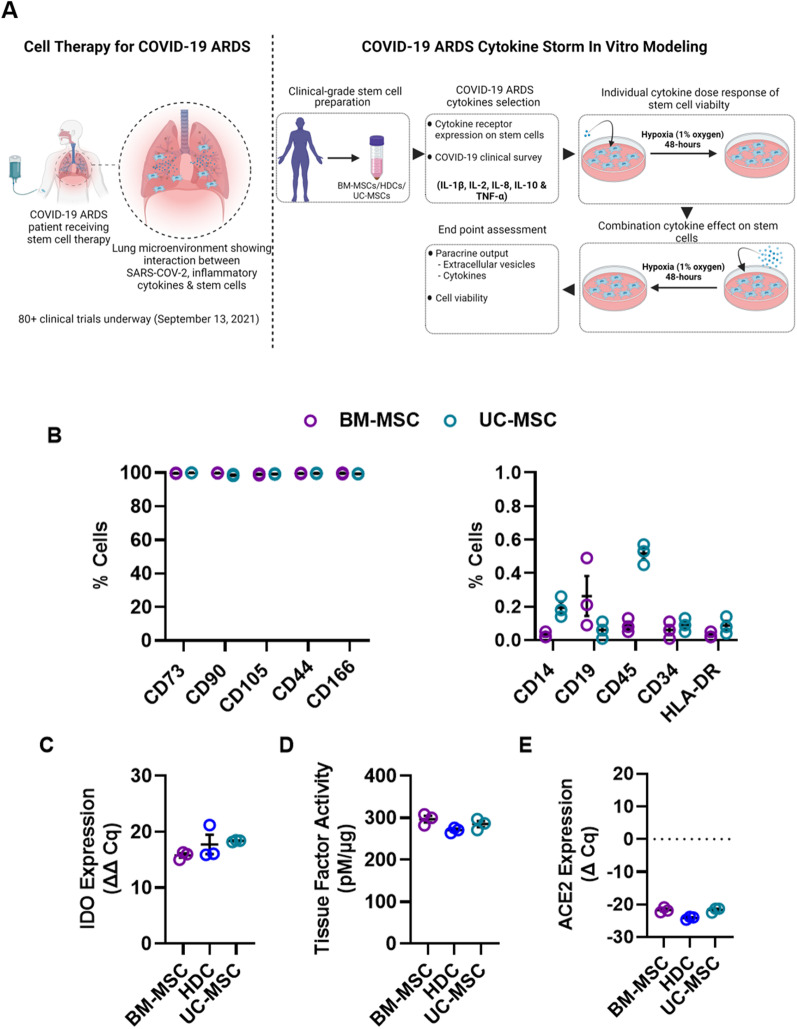
GMP stem cell manufacturing and characterization**. A** Conceptual figure showing COVID-19 ARDS patient lung microenvironment and methodological approach utilized in the study: All three cell products (BM-MSCs, HDCs, & UC-MSCs) were prepared to the clinical-grade standards in the cell manufacturing facility. Using COVID-19 clinical literature survey and flow cytometry cytokine receptor expression profile, five COVID-19 ARDS related cytokines (IL-1β, IL-2, IL-8, IL-10, and TNF-α) were selected for experimentation in the current study. Followed by an assessment of individual cytokine dose response on cell viability, the impact of cytokine cocktail containing all five cytokines at clinically relevant doses on paracrine output (extracellular vesicle production) and cell viability of three cell types was evaluated. All experiments were conducted at hypoxia (1% oxygen) for 48-h to simulate transplanted cell residence in hypoxic COVID-19 ARDS lung microenvironment. **B** Flow cytometry analysis showed BM-MSCs & UC-MSCs were positive for markers of mesenchymal stromal cell origin (CD73, CD90, CD105, CD166, and CD44) and negative for markers of hematopoietic cell origin (CD14, CD19, CD34, CD45 and HLA-DR). **C** qPCR showed IFN-γ treatment for 24-h induces IDO-gene expression in all three cell types (GAPDH was used as a reference gene). **D** Colorimetric assay showed Tissue factor activity of all three cell types was found to be similar. **E** qPCR showed none of the cell types express ACE2 (ΔCq values < -20 when normalized to reference gene, GAPDH). All data are presented as individual and mean values ± SEM, *n* = 3 biological replicates; each circle represents one data point from one unique biological replicate

Clinical grade BM-MSCs were prepared from BM donated by healthy volunteers enrolled in CISS-1 trial [25]. In collaboration with the Centre for Regenerative Therapies in Dresden, we cultured UC-MSCs from primary UC cell isolates [36]. Both BM-MSCs and UC-MSCs fulfilled standard flow cytometry definitions for MSCs (CD73, CD90, CD105, CD166, and CD44) and were negative for markers of hematopoietic contamination (CD14, CD19, CD34, CD45 and HLA-DR; Fig. 1B). As previously outlined, the BM- and UC-MSC cells used in this study exhibited tri-lineage (osteogenic, adipogenic, and lipogenic) differentiation [36, 37].

HDCs were cultured from atrial appendages in a cell manufacturing facility under constant physiological conditions using serum-free xenogen-free culture conditions. Given previous work characterizing the surface antigen markers of these CD105 + CD45- cells, HDC characterization was not repeated in this study [26].

Pre-treatment with IFN-γ enhanced IDO gene similarly in all 3 cell products (Fig. 1C). Given concerns regarding the emerging role of thrombosis in COVID-19 ARDS outcomes [38], we assessed TF activity which was similar in all 3 cell types (250–310 pM per µg of cell lysate; Fig. 1D). Finally, recent work has shown that ACE2 is involved in SARS-COV-2 endocytosis which raises concerns that ACE2 + cells could be susceptible to adverse effects from COVID-19 or provide a reservoir for viral sequestration [39]. Reassuringly, qPCR analysis showed that none of the cell types expressed ACE2 (ΔCq values < − 20 when normalized to reference gene, GAPDH; Fig. 1E); suggesting these cells would not be directly impacted by or sequester the virus.

### Bioactive molecules produced by cell products

**Fig. 2:**
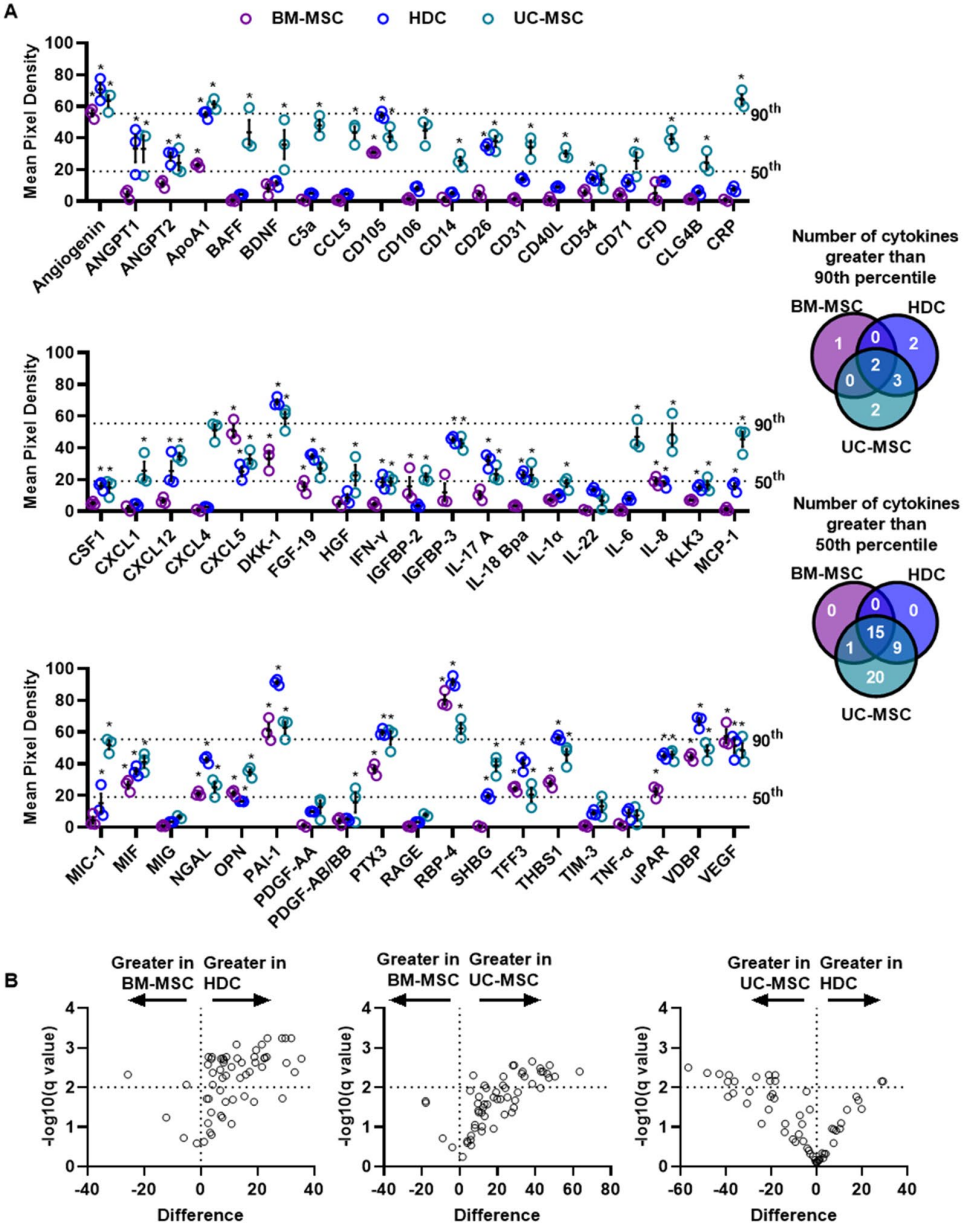
Cytokine profiling within media conditioned by cell products. **A** Proteomic profiling showing differentially expressed cytokines within media conditioned by cell products. The 50th and 90th percentile marks for mean pixel density are indicated to visualize cytokine abundance. Further, Venn diagram was used to show the number of cytokines that are distinct or common among cell types. Both HDCs and UC-MSCs showed robust cytokine production in comparison to BM-MSCs. Data were compared to baseline using Two-way ANOVA testing followed by with post hoc testing using Dunnet’s Multiple Comparisons test to determine the group(s) with the difference(s). *p < 0.05 compared to baseline. All data are presented as individual and mean values ± SEM, *n* = 3 biological replicates; each circle represents one data point from one unique biological replicate. **B** Volcano plots demonstrating the relationship between difference in cytokine expression by each cell type and the False Discovery Rate (i.e., the *q* value). HDCs produced 37 more cytokines vs. BM-MSCs (only 1 cytokine found to be greater in BM-MSCs). UC-MSCs produced 19 more cytokines vs. BM-MSCs and UC-MSCs produced 10 cytokines in greater abundance vs. HDCs (only 2 cytokines in greater abundance in HDCs). Data were compared using an unpaired *t*-test with individual variances for samples and a two-stage step-up (Benjamini, Krieger, and Yekutieli) false discovery rate to account for multiple comparisons

The salutary effects of transplanted adult cells are largely mediated by paracrine transfer of bioactive molecules, such as cytokines or EVs. As such, we explored the cytokines and EVs produced by all 3 cell types in hopes of identifying the most effective therapy for COVID-19 lung injury [26, 31, 32]. As shown in Fig. 2A and Additional file 2: Fig. S2, 57 of the 105 proteins assayed in the membrane-based sandwich immunoassay were secreted above baseline by at least one of the 3 cell types. Both HDCs and UC-MSCs provided robust cytokine production as depicted by the large number of cytokines found in the 50^th^ and 90^th^ percentile of mean pixel density compared to BM-MSCs. Significant differences in the cytokine repertoire produced by each cell type were also evident. When compared to BM-MSCs (Fig. 2B and Additional file 2: Fig. S2), HDCs produced 37 proteins that showed higher levels while only 1 cytokine was found to be greater (C-X-C Motif Chemokine Ligand 5, CXCL5) in BM-MSCs. When UC-MSCs were compared to BM-MSCs, they produced 19 cytokines that showed higher levels (Fig. 2B and Additional file 2: Fig. S3). When HDCs were compared directly to UC-MSCs, UC-MSCs produced 10 cytokines in greater abundance while HDCs produced 2 cytokines in greater abundance (Fig. 2B and Additional file 2: Fig. S4). All cytokines in the conditioned media of all 3 cell types are implicated in proliferation, wound healing, or immunomodulation, highlighting the potential therapeutic utility of these cells in COVID-19 ARDS.

**Fig. 3:**
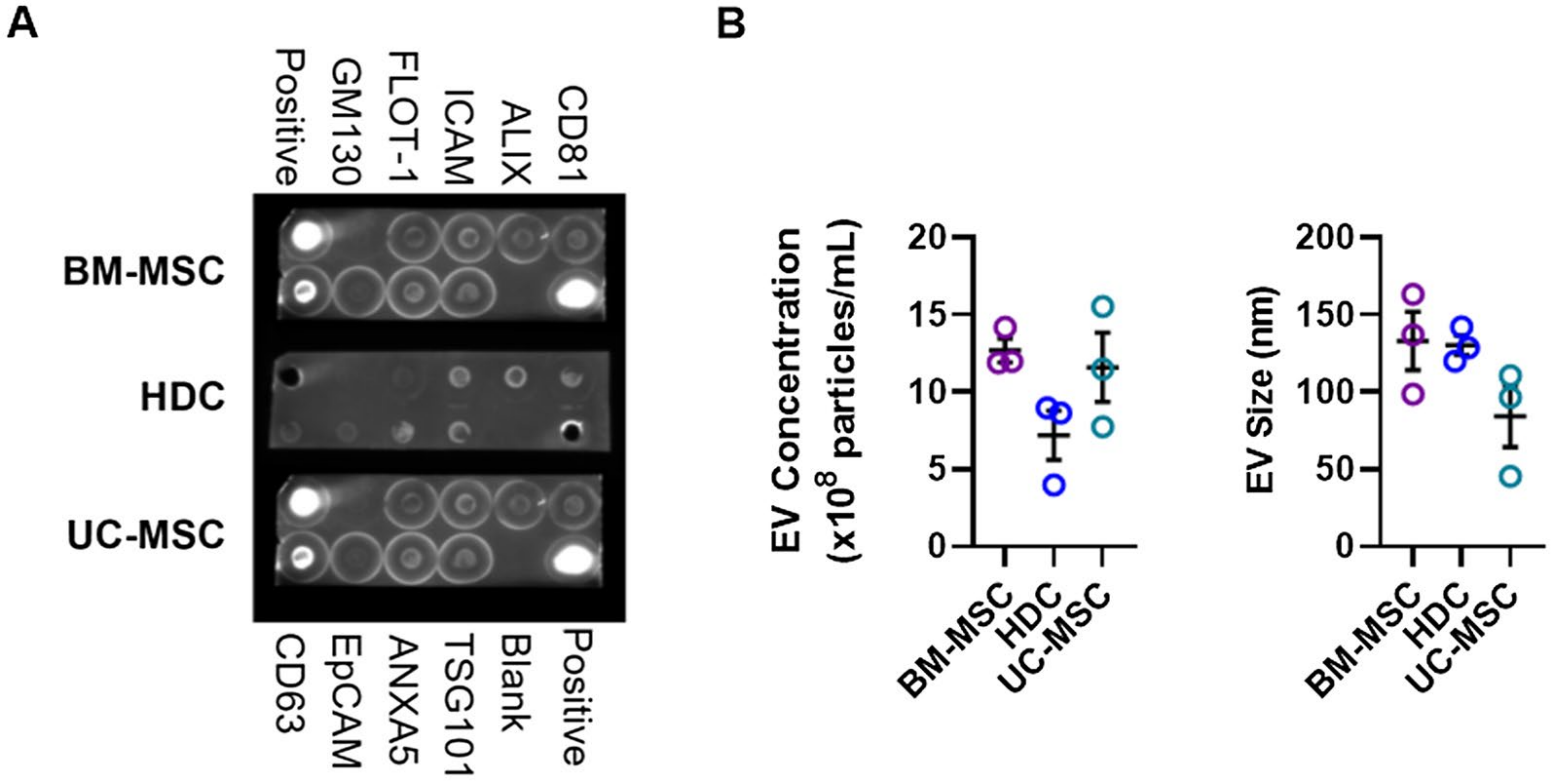
Extracellular vesicle (EV) profiling within media conditioned by cell products**. A** Proteomic antibody array showed the presence of 8 known EV markers (ICAM, ALIX, CD81, CD63, EPCAM, ANXAS, TSG101, FLOT-1) and the absence of bands for cis-Golgi marker (GM130) suggesting that EV preparations were free of cellular contaminants. **B** Nanoparticle tracking analysis showed the concentration and size of EVs isolated from all 3 cell types were representative of accepted definitions for EV identity and no significant differences were found between cell products. All data are presented as individual and mean values ± SEM, *n* = 3 biological replicates; each circle represents one data point from one unique biological replicate. Differences between cell types were analyzed by One-way ANOVA

EVs were isolated from conditioned media using differential ultracentrifugation [32]. As shown in Fig. 3A, all 3 isolates expressed surface markers commonly found in EVs (ICAM, ALIX, CD81, CD63, EPCAM, ANXAS, TSG101, FLOT-1) without markers indicative of cellular contamination (GM130). The concentration and size of EVs isolated from all 3 producer cell products were representative of accepted definitions for EV identity [40] with no significant differences detected between the cell products (Fig. 3B). Taken as a whole, this data indicates that all cell types produced large amounts of pro-healing bioactive molecules with the greatest abundance seen in UC-MSCs.

### Expression of receptors for cytokines implicated in COVID-19 ARDS

Given that transplanted cells will likely behave very differently in the ARDS lung as compared to basal media conditions, a search of the COVID-19 ARDS literature was performed to identify the cytokines implicated in acute lung injury (Additional file 1: Tables S2, S3). Although this search revealed several publications that described cytokine involvement in COVID-19, most of the published work focused on measuring blood cytokine levels. At the time of our literature search (January 1, 2021), we found 7 original research publications that reported cytokine levels in the lungs (i.e., bronchial alveolar lavage fluid) of COVID-19 patients (Additional file 1: Table S3 and Additional file 2: Fig. S5) [21–23, 41–44]. From this search, we identified 6 cytokines that are critically involved in the pathogenesis of COVID-19 induced ARDS lung injury (IL-1β, IL-2, IL-6, IL-8, IL-10, and TNFα).

**Fig. 4:**
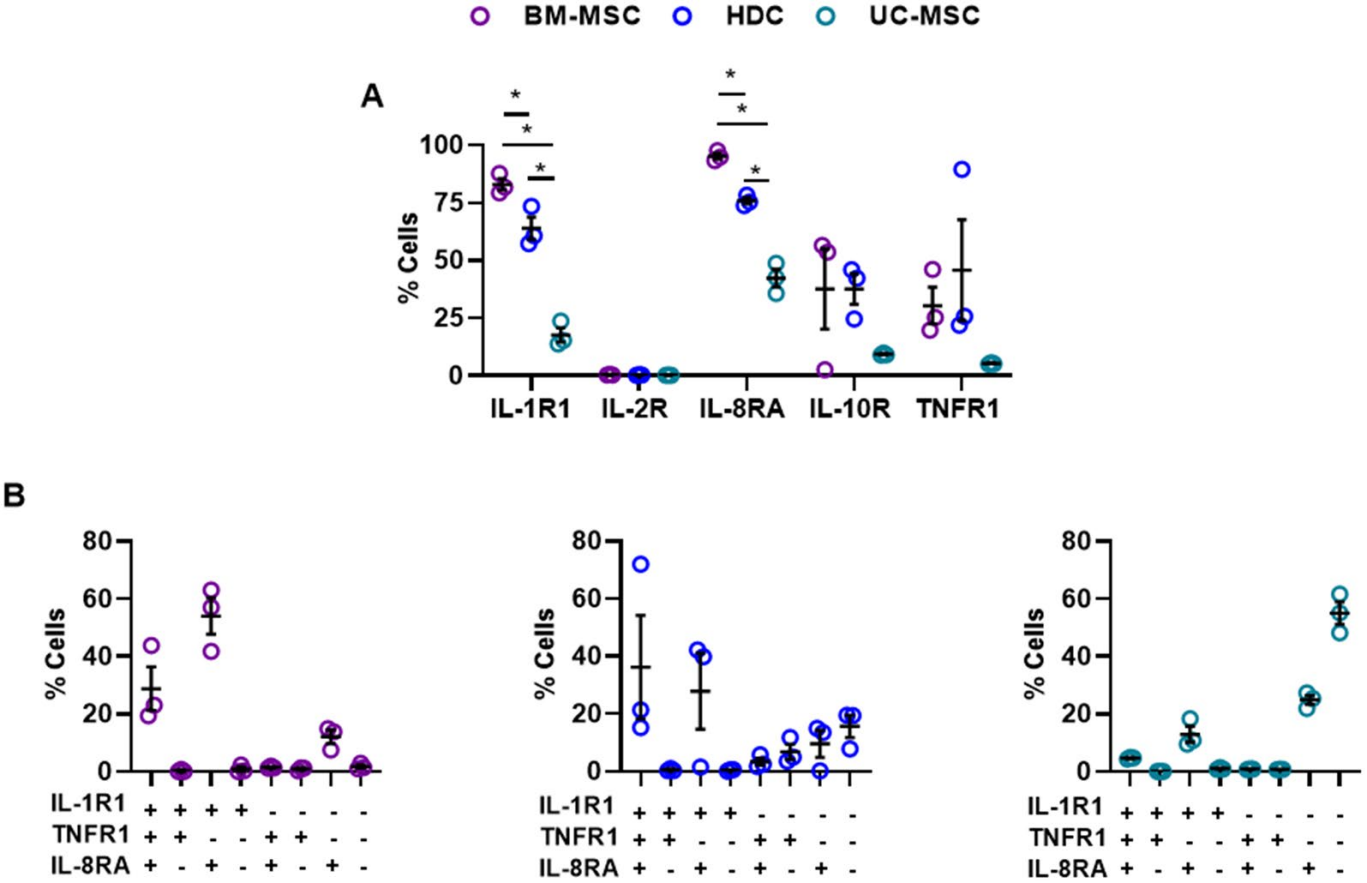
Receptor expression of COVID-19 ARDS associated cytokines on the cell products. **A** Flow cytometry analysis demonstrated all cell types express surface receptors for cytokines implicated in the COVID-19 ARDS pathology (except for IL-6); table showing the mean percentage cells that expressed cytokine receptors. Receptors for IL-1β or IL-8 were found to be expressed at a higher percentage in all cell types, while receptor for IL-2 was found to be minimally expressed. All data were analyzed by One-way ANOVA followed by post hoc testing using Tukey’s Multiple Comparisons test with *p ≤ 0.05 as indicated. **B** Flow cytometry co-expression analysis on three relatively highly expressed cytokine receptors showed that higher proportion of BM-MSCs and HDCs express COVID-19 ARDS cytokine receptors as compared to UC-MSCs. All data are presented as individual and mean values ± SEM, *n* = 3 biological replicates; each circle represents one data point from one unique biological replicate

Flow cytometry was used to profile the expression of receptors for the selected 6 cytokines implicated in COVID-19 ARDS (Fig. 4A; Additional file 2: Fig. S6). None of the cells expressed a receptor for IL-6; therefore, it was excluded from further experimentation. IL-1R1 and IL-8RA were found on many cells, while IL-2R was infrequently expressed (< 1%). To outline relevant cytokine responsive subpopulations, each cell type was profiled based on the 3 highly abundant cytokine receptors (IL-1R1, IL-8RA and TNFR1; Fig. 4B). Flow cytometry revealed that 36 ± 18% of HDCs were IL-1R1 +/IL-8RA +/TNFR1 + while 28 ± 13% were IL-1R1 +/IL-8RA + and very few HDCs expressed IL-1R1 alone (0.4 ± 0.2%). IL-1R1 +/IL-8RA +/TNFR1- cells were found in 54 ± 6% of BM-MSCs while 29 ± 7% were IL-1R1 +/IL-8RA +/TNFR1 +. Akin to HDCs, very few BM-MSCs that expressed only IL-1R1 (0.8 ± 0.7%; Fig. 4B). UC-MSCs expressed the fewest cytokine receptors for COVID-19. Only 4.7 ± 0.2% of cells were IL-1R1 +/IL-8RA +/TNFR1 + while 55 ± 4% were IL-1R1-/ IL-8RA-/TNFR1-. As such, BM-MSCs and HDCs contained notable subpopulations that expressed receptors for cytokines implicated in COVID-19 ARDS as compared to UC-MSCs; suggesting these cell products may be more susceptible to the adverse effects of COVID-19.

### Effect of individual COVID-19 ARDS cytokines on cell viability

To simulate the conditions found in COVID-19 ARDS lungs, we performed our experiments in basal media with COVID-19 ARDS cytokines under hypoxic (1% oxygen) conditions. From the literature search outlined above, we targeted relevant cytokine concentrations reported in COVID-19 patient lungs (IL-1β: 564 pg/mL, IL-2: 23 pg/mL, IL-8: 8661 pg/mL, IL-10: 131 pg/mL and TNF-α: 34 pg/mL). Given that flow cytometry showed all 3 cell products did not express a receptor for IL-6, it was excluded from experimentation.

**Fig. 5:**
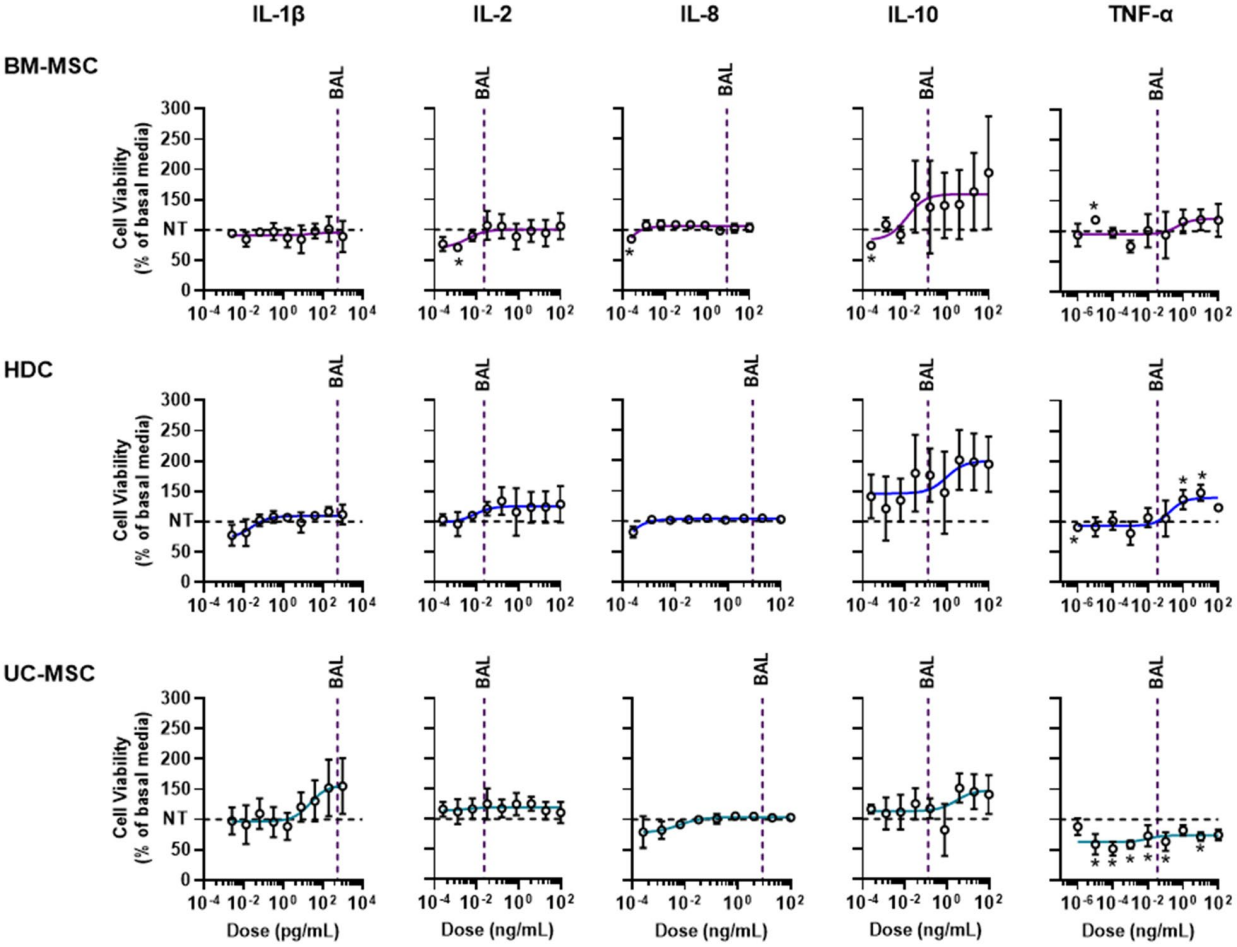
Biological activity of individual cytokines implicated in COVID-19 ARDS on cell products. Dose response assays showing the effect of cytokines on cell viability. All data are presented as mean ± SEM, *n* = 3 biological replicates; each circle represents one data point from one unique biological replicate. Data were analyzed using student’s *t* test with *p ≤ 0.05 as compared to NT as indicated. The cytokine doses were log transformed, and dose response curves were fitted using nonlinear regression (log cytokine vs response; three parameters) in GraphPad Prism v. 9.1

Cells were exposed to basal media with each individual cytokine at 9 escalating doses for 48 h prior to quantifying viability. The dose range was chosen to reflect at least one concentration tenfold greater than the largest bronchial alveolar lavage (BAL) fluid concentration recorded in the emerging COVID-19 ARDS literature. As shown in Fig. 5, BM-MSCs were largely unaffected by COVID-19 cytokines excepting minor variability at low non-clinical doses. HDC viability remained similarly unaffected excepting TNF-α at doses that were at least 100-fold greater than BAL specimens (i.e., 10 or 100 ng/mL) which increased proliferation. UC-MSC viability was not altered by exposure to IL-1β, IL-2, IL-8, and IL-10 while TNF-α at doses ~ 3000 times lower and ~ 3 times higher than BAL specimens markedly reduced viability. Collectively, these data indicate that individual cytokines had little effect on cell viability excepting TNF-α induced cytotoxicity in UC-MSCs.

### Impact of multiple simultaneous COVID-19 ARDS cytokines on cell viability and paracrine ability

**Fig. 6:**
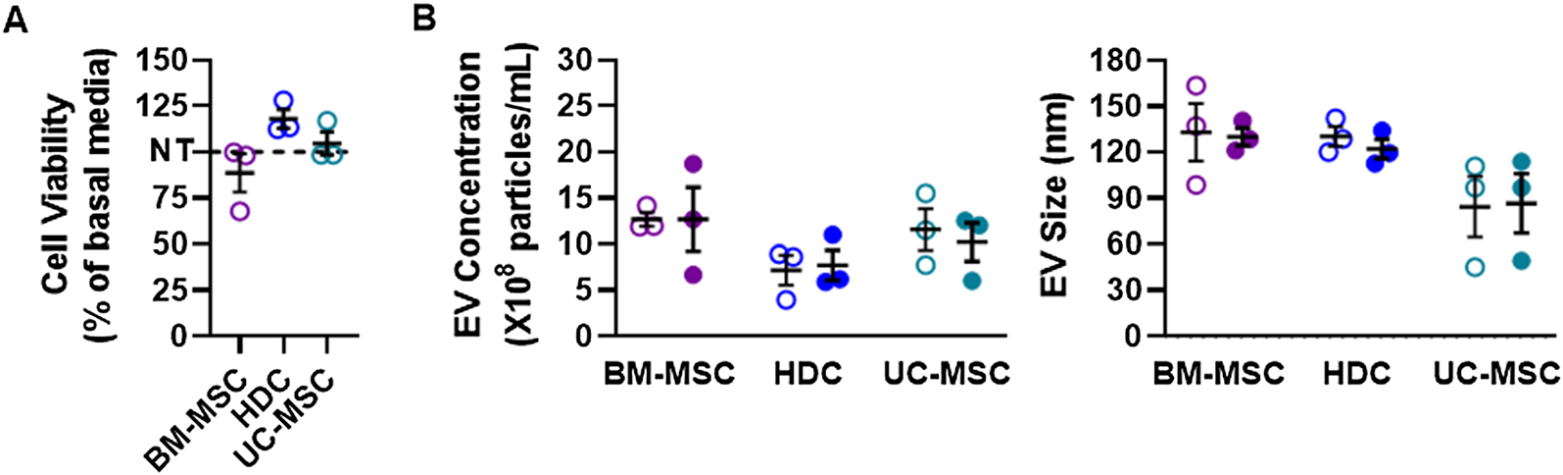
Cytokine cocktail treatment on cell viability and EV yield or size. **A** Cell viability assay showed that treating cell products with COVID-19 ARDS cytokine cocktail does not affect cell viability. All data are presented as percentage of no cytokine cocktail treatment group (basal media). **B** Nanoparticle tracking analysis demonstrated that cytokine cocktail does not affect either concentration or size of EVs produced by any of the cell types. Filled circles represent cytokine cocktail treatment, Hollow circles represent no cytokine cocktail treatment. All data are presented as individual and mean values ± SEM. *p ≤ 0.05 as indicated, *n* = 3 biological replicates; each circle represents one data point from one unique biological replicate

To evaluate the influence of the multiple cytokines seen in COVID-19 ARDS on cell viability and EV yield, cells were exposed all 5 cytokines at the concentrations reported in BAL fluid from critically ill COVID-19 patients. As shown in Fig. 6, simultaneous exposure to cytokines did not alter cell viability or EV yield and phenotype. In line with the individual cytokine biological activity data, these findings provide reassurance that the cytokines seen in critically ill patients with COVID-19 ARDS do not alter viability or the paracrine secretory ability of these cells currently under investigation for COVID-19 ARDS.

### Effect of COVID-19 ARDS cytokines on pulmonary endothelial permeability

To test the notion that COVID-19 cytokines may alter the functional effects of transplanted cells, we explored the effect of multiple simultaneous COVID-19 ARDS cytokines on UC-MSC effects on pulmonary endothelial permeability. As shown in Additional file 2: Fig. S7, incubating HPMECs with media conditioned by UC-MSCs treated with all 5 COVID-19 ARDS cytokines did not alter cell permeability.

## Discussion

Cell therapy for COVID-19 ARDS seeks to interrupt the toxic cytokine cascade initiated by COVID-19 infection. Most patients with severe COVID-19 lung injury exhibit cytokine release syndrome as pro-inflammatory mediators are produced within the lungs to impair local and distal tissues [4, 6]. In theory, this dysregulated immune response may interfere with cell therapies by boosting transplanted cell clearance or altering the pro-healing paracrine factors released by transplanted cells. Despite work done to initiate more than 80 clinical trials administering therapeutic immunomodulatory cells to patients with COVID-19 ARDS, no study to date has evaluated the impact of COVID-19 induced cytokines on cell therapy candidates. Hence, our study was undertaken to evaluate if the cytokines seen in COVID-19 has any effect on the 3 leading cell products (BM-MSCs, HDCs and UC-MSCs) currently under clinical evaluation.

Based on the COVID-19 cytokine literature, we identified 6 cytokines (IL-1β, IL-2, IL-6, IL-8, IL-10, and TNF-α) commonly found in critically ill COVID-19 patients [26, 31, 32]. These patients were targeted as most clinical trials investigating the effect of cell therapy on COVID-19 to date have elected to enroll only patients requiring mechanical ventilation. Ambulatory patients likely have a much different cytokine profile as early disease ensues, but this remains to be established. We focused on cytokines identified using BAL specimens rather than serum samples. BAL provides a highly sensitive and very specific measure of inflammatory markers within the lung. Often, these local increases can be independent of systemic inflammatory response syndromes [45]. Given that intravenous injected cells will collect within the lung to modify disease activity [46], we posited that testing cytokines at levels seen in BALs would reflect in part the conditions tested in clinical trials.

As shown in the results, clinical grade cell products expressed many of the receptors for cytotoxic cytokines seen in severe COVID-19 lung infection but withstood the toxic inductive effect of these cytokines at: 1) doses at least twofold greater than observed clinically, or 2) in combination at clinically relevant levels. These findings speak to the anti-apoptotic and anti-cell death reserves found in the cell products. Albeit initially profiled in cancer stem cells, adult and neonatal stem cells resist extrinsic cell death signalling through suppression of pro-apoptotic response elements and upregulation of anti-apoptotic proteins [47, 48]. Although the results of upcoming clinical trials are not yet known, our results would indicate that measures designed to increase transplanted cell resistance to adverse cytokine signalling would not alter treatment outcomes.

These findings led us to speculate on which cell therapy might provide the most appropriate response to a rising pandemic case load. Securing enough starting material to culture heart derived cells would be challenging in a pandemic setting as cell manufacturing is inherently tied to cardiac surgery case loads (atrial appendage harvest), the ability to schedule invasive ventricular biopsies or the availability of donor hearts that are not used for cardiac transplantation. Adult bone marrow isolation is feasible in a pandemic setting as this low-risk procedure could be safely performed if adequate distancing and safety measures are implemented. But umbilical cords may provide the optimal tissue source as screened cords are readily available around the world, even during pandemic peaks. Given that UC-MSCs also produced the largest number of cytokines implicated in proliferation, wound healing and immunomodulation, our study would indicate that UC-MSCs provide the most available cell type with the largest pro-healing paracrine repertoire to treat patients with severe COVID-19 lung injury.

Despite these findings, our study has numerous limitations which include: (1) Untested cytotoxic cytokines. Many of the studies that profiled COVID-19 ARDS focused on candidate screening for cytokines implicated in other forms of ARDS. Unbiased proteomic profiling of BAL specimens from COVID 19 ARDS has yet to be performed. As such, our cytokine cocktail may have missed important cytotoxic cytokines that would influence transplanted cell survival and function. (2) Cytotoxic extracellular vesicles. Recent evidence has shown that sera from patients with severe COVID-19 patients is enriched with sphingomyelins and monosialodihexosyl ganglioside elements [49]. Importantly, the latter correlates with disease severity and may play a role in transplanted cell survival/paracrine production which was not tested in this study. (3) Lack of in vivo testing. Implementing a true animal model for COVID-19 ARDS is challenging, particularly in times of strained resources with competing technologies (i.e., vaccine development) [50, 51]. Similarly, the extent to which historical models of ARDS (such as lipopolysaccharide or non-COVID infectious pathogen-induced ARDS) [9, 52, 53] simulate COVID-19 lung injury is not known. As such, we chose to use a reductionist approach to model the direct effect of known cytokines on uniform measures of cell performance and viability. Future comparisons using in vivo model testing may be needed to see how these findings hold up to scrutiny when robust animal models of COVID-19 ARDS are available. (4) COVID-19 cytokine effects on paracrine production. Given that the cytokines produced by COVID-19 had limited effects on cell viability or other measures of function, the influence of pathway stimulation on candidate cell cytokine production was likely negligible and not profiled. Future work might be directed towards profiling these effects but should be performed in animal models of COVID-19 ARDS to enhance translational utility.

## Conclusions

Our findings suggest that the pro-inflammatory cytokines seen in patients with COVID-19 ARDS will not impair the survival and paracrine ability of candidate cell products. These new findings will inform on the interpretation of ongoing efficacy trials and bolster efforts at developing new cellular therapies for patients with COVID-19 ARDS.

## Supplementary Information

**Additional file 1.** Based on analysis of the litterature, a range of cytokine doses was chosen and performed.

**Additional file 2.Fig. S1**. Cytokine profiling in conditioned media produced by potential COVID-19 cell products. Representative images of the proteome array used to analyze conditioned media produced by BM-MSCs, HDCs, or UC-MSCs. The table on the right panel lists the position coordinates of cytokine antibody on the array.**Fig. S2** Relative cytokine content within HDC conditioned media compared to BM-MSC conditioned media. Expression of cytokines within conditioned media produced by HDCs compared to BM-MSCs. Data were compared using an unpaired t-test with individual variances for samples and a two-stage step-up (Benjamini, Krieger, and Yekutieli) false discovery rate to account for multiple comparisons. All data are presented as individual and mean values ± SEM, n=3 biological replicate; each circle represents one data point from one unique biological replicate. Significance is indicated on the panels. **Fig. S3**. Relative cytokine content within UC-MSC conditioned media compared to BM-MSC conditioned media. Expression of cytokines within conditioned media produced by UC-MSCs compared to BM-MSCs. Data were compared using an unpaired t-test with individual variances for samples and a two-stage step-up (Benjamini, Krieger, and Yekutieli) false discovery rate to account for multiple comparisons. All data are presented as individual and mean values ± SEM, n=3 biological replicates; each circle represents one data point from one unique biological replicate. Significance is indicated on the panels.**Fig. S4**. Relative cytokine content within UC-MSC conditioned media compared to HDC conditioned media. Expression of cytokines within conditioned media produced by UC-MSCs compared to HDCs. Data were compared using an unpaired t-test with individual variances for samples and a two-stage step-up (Benjamini, Krieger, and Yekutieli) false discovery rate to account for multiple comparisons. All data are presented as individual and mean values ± SEM with n=3 biological replicates; each circle represents one data point from one unique biological replicate. Significance is indicated on the panels. **Fig. S5**. Cytokine concentrations found within the lung of critically ill COVID-19 patients. This figure depicts literature search results of cytokine concentrations found in bronchial alveolar lavage fluid from critically ill patients with COVID-19. When data were not presented in a tabular form, they were extracted from the figures by using online tool WebPlot Digitizer. All data are presented as individual and mean values ± SEM. The values on the bar graphs represent arithmetic mean of all studies for respective cytokine. **Fig. S6**. Flow cytometry showing co-segregation of receptors for COVID-19 related cytokines. Representative flow cytometry images demonstrating detection of FITC anti-human IL-1R1, BV421 anti-human IL-2Rβ, PerCP-eFluor710 anti-human IL-6R, PerCP-cy5.5 anti-human IL-8RA, APC anti-human IL-10R, and PE anti-human TNFR1on BM-MSCs, HDCs and UC-MSCs. Isotype controls were used to correct compensation and confirm antibody specificity.**Fig. S7**. COVID-19 ARDS cytokine challenged UC-MSC conditioned media effect on pulmonary microvascular endothelial cell permeability. Endothelial cell permeability assay showed that treating pulmonary microvascular endothelial cells with conditioned media (CM) collected from COVID-19 ARDS cytokine cocktail challenged UC-MSCs does not alter endothelial cell permeability. Filled circles represent cytokine cocktail treatment, Hollow circles represent no cytokine cocktail treatment. All data are presented as individual and mean values ± SEM. n=3 biological replicates; each circle represents one data point from one unique biological replicate.

## Data Availability

The data are available from the corresponding author on reasonable request.

## Abbreviations

ACE2: Angiotensin converting enzyme 2
ALIX: Apoptosis-linked gene 2–interacting protein
ANGPT: Angiopoietin
ANXA5: Annexin A5
ApoA1: Apolipoprotein A1
ARDS: Acute respiratory distress syndrome
BAFF: B-cell activating factor
BAL: Bronchiolar alveolar lavage
BDNF: Brain derived neurotrophic factor
BM-MSC: Bone marrow derived mesenchymal stromal cells
C5a: Complement component 5a
CD: Cluster of differentiation
CFD: Complement factor D
CLG4B: Collagenase type IV B
CRP: C-reactive protein
CSF: Colony stimulating factor
CXCL/CCL: Chemokine ligand
DKK: Dickkopf
EpCAM: Epithelial cell adhesion molecule
EV: Extracellular vesicles
FGF: Fibroblast growth factor
FLOT1: Flotillin-1
GM130: Cis-Golgi matrix protein
HDC: Heart derived cells
HGF: Hepatocyte growth factor
HLA-DR: Human leukocyte antigen-DR
ICAM1: Intercellular adhesion molecule 1
IDO: Indoleamine-2,3-dioxygenase
IFN-γ: Interferon-gamma
IGFBP: Insulin-like growth factor binding protein
IL: Interleukin
IL-10R: Interleukin-10 receptor
IL-1R: Interleukin-1β receptor
IL-2R: Interleukin-2 receptor
IL-8RA: Interleukin-8 receptor
KLK3: Kallikrein 3
MCP: Monocyte chemoattractant protein
MIC: Macrophage inhibitory cytokine
MIF: Macrophage migration inhibitory factor
MIG: Monokine induced by interferon-γ
NGAL: Neutrophil gelatinase-associated lipocalin
ns: Non significant
NT: No treatment
OPN: Osteopontin
PAI: Plasminogen activator inhibitor
PDGF: Platelet derived growth factor
PTX3: Pentraxin 3
RAGE: Receptor for advanced glycation end products
RBP: Retinol-binding protein
SARS-COV-2: Severe acute respiratory syndrome coronavirus 2
SHBG: Sex hormone binding globulin
TFF3: Trefoil factor 3
THBS1: Thrombospondin-1
TIM: T-cell immunoglobulin and mucin domain-containing protein
TNF: Tumor necrosis factor
TNFR1: Tumor necrosis factor receptor 1
TSG101: Tumor susceptibility gene 101
UC-MSC: Umbilical cord derived mesenchymal stromal cells
uPAR: Urokinase plasminogen activator
VDBP: Vitamin-D binding protein
VEGF: Vascular endothelial growth factor
