# Supplementary material for: Direct comparison of different therapeutic cell types susceptibility to inflammatory cytokines associated with COVID-19 acute lung injury
Source: Stem Cell Res Ther. 2022 Jan 15;13:20. doi: 10.1186/s13287-021-02699-7 (PMC8760881; doi:10.1186/s13287-021-02699-7)
Supplement: Supplementary file 1 — Additional file 1. Based on analysis of the litterature, a range of cytokine doses was chosen and performed. [file 13287_2021_2699_MOESM1_ESM.docx]

**Table S1. List of Stem Cell based clinical trials for COVID-19 ARDS (clinicaltrials.org, accessed September 14, 2021).**

| Intervention/Cell type | NCT# | Status | Phase | Sponsor/Location |
| --- | --- | --- | --- | --- |
| Umbilical Cord-Derived MSCs | NCT04416139 | Recruiting | Phase 2 | Instituto Nacional de Ciencias Medicas y Nutricion Salvador Zubiran/ Mexico |
| Wharton's Jelly-Derived MSCs | NCT04390152 | Recruiting | Phase 1 & 2 | BioXcellerator/ Colombia |
| MSCs | NCT04366063 | Recruiting | Phase 2 & 3 | Royan Institute/ Iran |
| Wharton's Jelly- Derived MSCs | NCT04456361 | Active | Early Phase 1 | Instituto de Medicina Regenerativa/ Mexico |
| Umbilical Cord Blood-Derived MSCs | NCT04565665 | Recruiting | Phase 1 & 2 | M.D. Anderson Cancer Center/ USA |
| MSC-Derived Exosomes | NCT04798716 | Not yet recruiting | Phase 1 & 2 | AVEM HealthCare/ USA |
| MSCs | NCT04371393 | Active, not recruiting | Phase 3 | Icahn School of Medicine at Mount Sinai/ USA |
| Wharton's Jelly- Derived MSCs | NCT04625738 | Completed | Phase 2 | Central Hospital/ France |
| Umbilical Cord Tissue-Derived MSCs | NCT04490486 | Not yet recruiting | Phase 1 | University of Miami/ USA |
| CAStem | NCT04331613 | Recruiting | Phase 1 & 2 | Chinese Academy of Sciences/ China |
| Adipose-Derived MSCs | NCT04905836 | Recruiting | Phase 2 | Sorrento Therapeutics, Inc./ USA |
| Wharton's Jelly- Derived MSCs | NCT04390139 | Recruiting | Phase 1 & 2 | Banc de Sang i Teixits/ Spain |
| Umbilical Cord-Derived MSCs | NCT04355728 | Completed | Phase 1 & 2 | University of Miami/ USA |
| Human Umbilical Cord-Derived CD362 enriched MSCs | NCT03042143 | Active, not recruiting | Phase 1 & 2 | Belfast Health and Social Care Trust/ UK |
| Bone Marrow-Derived MSCs | NCT04377334 | Not yet recruiting | Phase 2 | University Hospital Tuebingen/ Germany |
| Adipose-Derived MSCs | NCT04903327 | Recruiting | Phase 2 | Sorrento Therapeutics, Inc./ USA |
| Bone Marrow MSC-Derived Extracellular Vesicles | NCT04493242 | Completed | Phase 2 | Direct Biologics, LLC/ USA |
| Bone Marrow-Derived MSCs | NCT04447833 | Active, not recruiting | Phase 1 | Uppsala University/ Sweden |
| Cymerus MSCs | NCT04537351 | Recruiting | Phase 1 & 2 | Cynata Therapeutics Limited/ Australia |
| MultiStem | NCT04367077 | Recruiting | Phase 2 & 3 | Athersys, Inc/ USA |
| Umbilical Cord MSCs | NCT04288102 | Completed | Phase 2 | Beijing 302 Hospital/ China |
| Umbilical Cord MSCs | NCT04269525 | Recruiting | Phase 2 | ZhiYong Peng, Zhongnan Hospital/ China |
| Umbilical Cord MSCs | NCT04457609 | Recruiting | Phase 1 | Indonesia University/ Indonesia |
| CAP-1002 (Cardiosphere Derived Cells) | NCT04623671 | Recruiting | Phase 2 | Capricor Inc./ USA |
| Umbilical Cord-Derived MSCs | NCT04400032 | Completed | Phase 1 & 2 | Ottawa Hospital Research Institute/ Canada |
| Umbilical Cord- Derived MSCs | NCT04865107 | Recruiting | Phase 2 | Ottawa Hospital Research Institute/ Canada |
| MSCs | NCT04713878 | Completed | Not applicable | Kanuni Sultan Suleyman Training and Research Hospital/ Turkey |
| Umbilical Cord- Derived MSCs | NCT04333368 | Active, not recruiting | Phase 1 & 2 | Assistance Publique - Hôpitaux de Paris/ France |
| Bone Marrow- Derived MSCs | NCT04445454 | Recruiting | Phase 1 & 2 | University of Liege/ Belgium |
| Adipose-Derived MSCs | NCT04348435 | Completed | Phase 2 | Hope Biosciences Stem Cell Research Foundation/ USA |
| Umbilical Cord MSCs | NCT04273646 | Not yet recruiting | Not applicable | Wuhan Union Hospital/ China |
| MSCs | NCT04252118 | Recruiting | Phase 1 | Beijing 302 Hospital/ China |
| Wharton's Jelly- Derived MSCs | NCT04313322 | Recruiting | Phase 1 | Stem Cells Arabia/ Jordan |
| MSCs | NCT04361942 | Recruiting | Phase 2 | Red de Terapia Celular/ Spain |
| Umbilical Cord MSCs | NCT04339660 | Recruiting | Phase 1 & 2 | Puren Hospital Affiliated to Wuhan University of Science and Technology/ China |
| Umbilical Cord MSCs | NCT04869397 | Recruiting | Phase 2 | McGill University Health Centre/ Canada |
| Bone Marrow- Derived MSCs | NCT04345601 | Recruiting | Phase 1 & 2 | Baylor College of Medicine/ USA |
| Adipose-Derived MSCs | NCT04611256 | Recruiting | Phase 1 | Hospital Reg. Lic. Adolfo Lopez Mateos/ Mexico |
| Bone Marrow- Derived MSCs | NCT05125562 | Recruiting | Phase 2 | Direct Biologics, LLC/ USA |
| Umbilical Cord MSCs | NCT05132972 | Recruiting | Phase 2 & 3 | Kementerian Riset dan Teknologi/ Indonesia |
| Olfactory Mucosa-Derived MSCs | NCT04382547 | Completed | Phase 1 & 2 | Institute of Biophysics and Cell Engineering of National Academy of Sciences of Belarus/ Belarus |
| MSCs | NCT04467047 | Not yet recruiting | Phase 1 | Hospital de Clinicas de Porto Alegre/ Brazil |
| MSC hypoxic secretome | NCT04753476 | Recruiting | Phase 2 | Stem Cell and Cancer Research Indonesia/ Indonesia |
| MSCs | NCT04525378 | Recruiting | Phase 1 | D'Or Institute for Research and Education/ Brazil |
| MSCs | NCT04444271 | Recruiting | Phase 2 | National Institute of Blood and Marrow Transplant (NIBMT), Pakistan |
| MSC secretome | NCT05122234 | Completed | Phase 3 | Indonesia University/ Indonesia |
| Bone Marrow- Derived MSCs | NCT04346368 | Not yet recruiting | Phase 1 & 2 | Guangzhou Institute of Respiratory Disease/ China |
| Bone Marrow- Derived MSCs | NCT04397796 | Active, not recruiting | Phase 1 | ImmunityBio, Inc./ USA |
| Adipose Tissue- Derived MSCs | NCT04527224 | Not yet recruiting | Phase 1 & 2 | Nature Cell Co. Ltd./ South Korea |
| Placenta-Derived MSCs | NCT04461925 | Recruiting | Phase 1 & 2 | Institute of Cell Therapy/ Ukraine |

**Table S2. Clinical literature survey of COVID-19 ARDS associated cytokines.**

| 1. **PubMed** | | |
| --- | --- | --- |
| **#** | **Query** | **Results** |
| 1 | (COVID 19[tw] OR COVID-19[tw] OR COVID- 19[tw] COVID19[tw] OR SARS CoV 2 Infection[tw] OR SARS-CoV-2*[tw] OR "COVID-19"[Mesh] OR "SARS-CoV-2"[Mesh]) | 119,501 |
| 2 | ("Cytokines"[Mesh] OR "Cytokine Release Syndrome"[Mesh] OR "Interleukin-1beta"[Mesh]OR "Interleukin-2"[Mesh] OR "Interleukin-6"[Mesh] OR "Interleukin-8"[Mesh] OR "Interleukin-10"[Mesh] OR "Tumor Necrosis Factor-alpha"[Mesh] OR Proinflammatory*[tw] OR Cytokine*[tw] OR Cytokine Storm*[tw] OR IL-1 beta[tw] OR Interleukin-1 beta[tw] OR IL-2[tw] OR Interleukine 2[tw] OR Interleukin 2[tw] OR Interleukin 6[tw] OR IL6[tw] OR IL-6[tw] OR Interleukin 8[tw] OR IL8[tw] OR IL-8[tw] OR CXCL8 Chemokine[tw] OR IL10[tw] OR IL-10[tw] OR Interleukin 10[tw] OR Tumor Necrosis Factor alpha[tw] OR TNFalpha[tw] OR TNF-alpha[tw]) | 116,104 |
| 3 | ("Body Fluids"[Mesh] OR "Lung"[Mesh] OR "Bronchoalveolar Lavage"[Mesh] OR Lungs[tw] OR Bronchoalveolar Lavage[tw] OR bronchoalveolar lavage fluid[tw] OR broncho-alveolar lavage fluid[tw] OR Lung Lavage[tw] OR Bronchopulmonary Lavage[tw] OR BALF[tw] OR body fluid*[tw]) | 38,347 |
| 4 | #1 AND #2 AND #3 AND (2019:2021[pdat])) | 626 |
| 5 | #4 FILTER/ONLY (Meta-Analysis, Review, Systematic Review) | 215 |
| 6 | #4 NOT #5 | 411 |

| 1. **Scopus** | | | | |
| --- | --- | --- | --- | --- |
| **#** | | **Searches** | **Results** | |
| 1 | | TITLE-ABS ("COVID 19" OR "COVID-19" OR "COVID- 19" OR "COVID19" OR "SARS CoV 2 Infection" OR "SARS-CoV-2*" OR "COVID-19" OR "SARS-CoV-2") | 130,585 | |
| 2 | | TITLE-ABS ("Cytokines" OR "Cytokine Release Syndrome" OR "Interleukin-1beta" OR "Interleukin-2" OR "Interleukin-6" OR "Interleukin-8" OR "Interleukin-10" OR "Tumor Necrosis Factor-alpha" OR "Proinflammatory*" OR "Cytokine*" OR "Cytokine Storm*" OR "IL-1 beta" OR "Interleukin-1 beta" OR "IL-2" OR "Interleukine 2" OR "Interleukin 2" OR "Interleukin 6" OR "IL6" OR "IL-6" OR "Interleukin 8" OR "IL8" OR "IL-8" OR "CXCL8 Chemokine" OR "IL10" OR "IL-10" OR "Interleukin 10" OR "Tumor Necrosis Factor alpha" OR "TNFalpha" OR "TNF-alpha") | 653,401 | |
| 3 | | TITLE-ABS ("Body Fluids" OR "Lung" OR "Bronchoalveolar Lavage" OR "Lungs" OR "Bronchoalveolar Lavage" OR "bronchoalveolar lavage fluid" OR "broncho-alveolar lavage fluid" OR "Lung Lavage" OR "Bronchopulmonary Lavage" OR "BALF" OR "body fluid*") | 860,846 | |
| 4 | | 1 AND 2 AND 3 | 884 | |
| 5 | | 4 AND PUBYEAR > 2018 AND NOT ( TITLE ( review ) ) AND NOT ( TITLE ( meta-analysis ) ) AND NOT ( TITLE ( "systematic review" ) ) AND ( LIMIT-TO ( DOCTYPE , "ar" ) ) | 508 | |
| 1. **Embase** | | | | |
| **#** | **Searches** | | | **Results** |
| 1 | (COVID 19 or COVID-19 or COVID19 or SARS CoV 2 Infection or SARS-CoV-2*).ti,ab,kw. | | | 116198 |
| 2 | coronavirus disease 2019/ | | | 100753 |
| 3 | exp severe acute respiratory syndrome coronavirus 2/ | | | 15258 |
| 4 | 1 or 2 or 3 | | | 123420 |
| 5 | exp cytokine/ | | | 1635672 |
| 6 | cytokine release syndrome/ | | | 3733 |
| 7 | (Proinflammatory* or Cytokine* or Cytokine Storm* or IL-1 beta or Interleukin-1 beta or IL-2 or Interleukine 2 or Interleukin 2 or Interleukin 6 or IL6 or IL-6 or Interleukin 8 or IL8 or IL-8 or CXCL8 Chemokine or IL10 or IL-10 or Interleukin 10 or Tumor Necrosis Factor alpha or TNFalpha or TNF-alpha).ti,ab,kw. | | | 858988 |
| 8 | 5 or 6 or 7 | | | 1823256 |
| 9 | body fluid/ or lung extravascular fluid/ or lung fluid/ | | | 37140 |
| 10 | exp lung/ | | | 401075 |
| 11 | lung lavage/ | | | 54721 |
| 12 | Lung? or Bronchoalveolar Lavage or broncho-alveolar lavage or Lung Lavage or Bronchopulmonary Lavage or BALF or body fluid*).ti,ab,kw. | | | 1123061 |
| 13 | 9 or 10 or 11 or 12 | | | 1241548 |
| 14 | 4 and 8 and 13 | | | 1490 |
| 15 | limit 14 to yr="2019 -Current" | | | 1490 |

The clinical literature search to identify cytokines implicated in COVID-19 ARDS pathology was performed using 3 separate search databases, 1) PubMed, 2) Scopus, 3) Embase. The search included only original research articles published from 2019-present.

**Table S3. Studies quantifying lung cytokine levels in COVD 19 patients.**

| **Author** | **PubMed ID** | **# of Patients** | **IL-1β** | **IL-2** | **IL-6** | **IL-8** | **IL-10** | **TNF-α** |
| --- | --- | --- | --- | --- | --- | --- | --- | --- |
| Ronit et al | 32979342 | 4 |  |  | 1080 | 3356 |  |  |
| Wang et al | 32422085 | 1 |  |  | 5763 |  | 45 |  |
| Liao et al | 32398875 | 13 | 1478 | 4 | 1840 | 4563 | 6 | 37 |
| Xu et al | 33101705 | 13 | 143 | 2 | 301 | 9608 | 2 | 8 |
| Pandolfi et al | 33198751 | 28 |  |  | 372 | 891 | 316 |  |
| Carvelli et al | 32726800 | 4 | 72 | 62 |  | 14492 |  | 56 |
| Wu et al | 33411411 | 26 |  |  |  | 19056 | 286 |  |
| **Mean cytokine concentration (pg/mL)** | | | **564** | **23** | **1871** | **8661** | **131** | **34** |

Based on the search results from Table S1, a total of seven original research articles that quantified lung cytokine levels were identified. All seven studies measured cytokine levels in bronchoalveolar lavage fluid (BALF) of COVID-19 patients and the concentrations reported represent an arithmetic mean of all the patients in a given study and are expressed in pg/mL. In studies where the cytokine data were not presented in a tabular form, the data were extracted from the figures by using online tool WebPlot Digitizer (<https://automeris.io/WebPlotDigitizer/>).

**Table S4. Cytokine dose response assay information**

| **Cytokine** | **Cat# (company)** | **Dose range (pg/mL or ng/mL)** | **Assay** | **Assay Readout** |
| --- | --- | --- | --- | --- |
| Recombinant Human IL-1 beta/IL-1F2 Protein | 201-LB-005 (R&D) | 0.00256-1000^a^ | CCK8 assay | Cell viability |
| Recombinant Human IL-2 Protein | 202-IL-010 (R&D) | 0.00026-100 | CCK8 assay | Cell viability |
| Recombinant Human IL-10 (aa 19-178) Protein | 1064-IL-010(R&D) | 0.00026-100 | CCK8 assay | Cell viability |
| Recombinant Human TNF-alpha Protein | 210-TA-005(R&D) | 0.000001-100 | CCK8 assay | Cytotoxicity |
| Recombinant Human IL-8/CXCL8 Protein | 208-IL-010/CF(R&D) | 0.00026-100 | CCK8 assay | Cell viability |

^a^ pg/mL
