# Supplementary material for: Direct comparison of different therapeutic cell types susceptibility to inflammatory cytokines associated with COVID-19 acute lung injury
Source: Stem Cell Res Ther. 2022 Jan 15;13:20. doi: 10.1186/s13287-021-02699-7 (PMC8760881; doi:10.1186/s13287-021-02699-7)
Supplement: Supplementary file 2 — Additional file 2.Fig. S1. Cytokine profiling in conditioned media produced by potential COVID-19 cell products. Representative images of the proteome array used to analyze conditioned media produced by BM-MSCs, HDCs, or UC-MSCs. The table on the right panel lists the position coordinates of cytokine antibody on the array.Fig. S2 Relative cytokine content within HDC conditioned media compared to BM-MSC conditioned media. Expression of cytokines within conditioned media produced by HDCs compared to BM-MSCs. Data were compared using an unpaired t-test with individual variances for samples and a two-stage step-up (Benjamini, Krieger, and Yekutieli) false discovery rate to account for multiple comparisons. All data are presented as individual and mean values ± SEM, n=3 biological replicate; each circle represents one data point from one unique biological replicate. Significance is indicated on the panels. Fig. S3. Relative cytokine content within UC-MSC conditioned media compared to BM-MSC conditioned media. Expression of cytokines within conditioned media produced by UC-MSCs compared to BM-MSCs. Data were compared using an unpaired t-test with individual variances for samples and a two-stage step-up (Benjamini, Krieger, and Yekutieli) false discovery rate to account for multiple comparisons. All data are presented as individual and mean values ± SEM, n=3 biological replicates; each circle represents one data point from one unique biological replicate. Significance is indicated on the panels.Fig. S4. Relative cytokine content within UC-MSC conditioned media compared to HDC conditioned media. Expression of cytokines within conditioned media produced by UC-MSCs compared to HDCs. Data were compared using an unpaired t-test with individual variances for samples and a two-stage step-up (Benjamini, Krieger, and Yekutieli) false discovery rate to account for multiple comparisons. All data are presented as individual and mean values ± SEM with n=3 biological replicates; each ci [file 13287_2021_2699_MOESM2_ESM.docx]

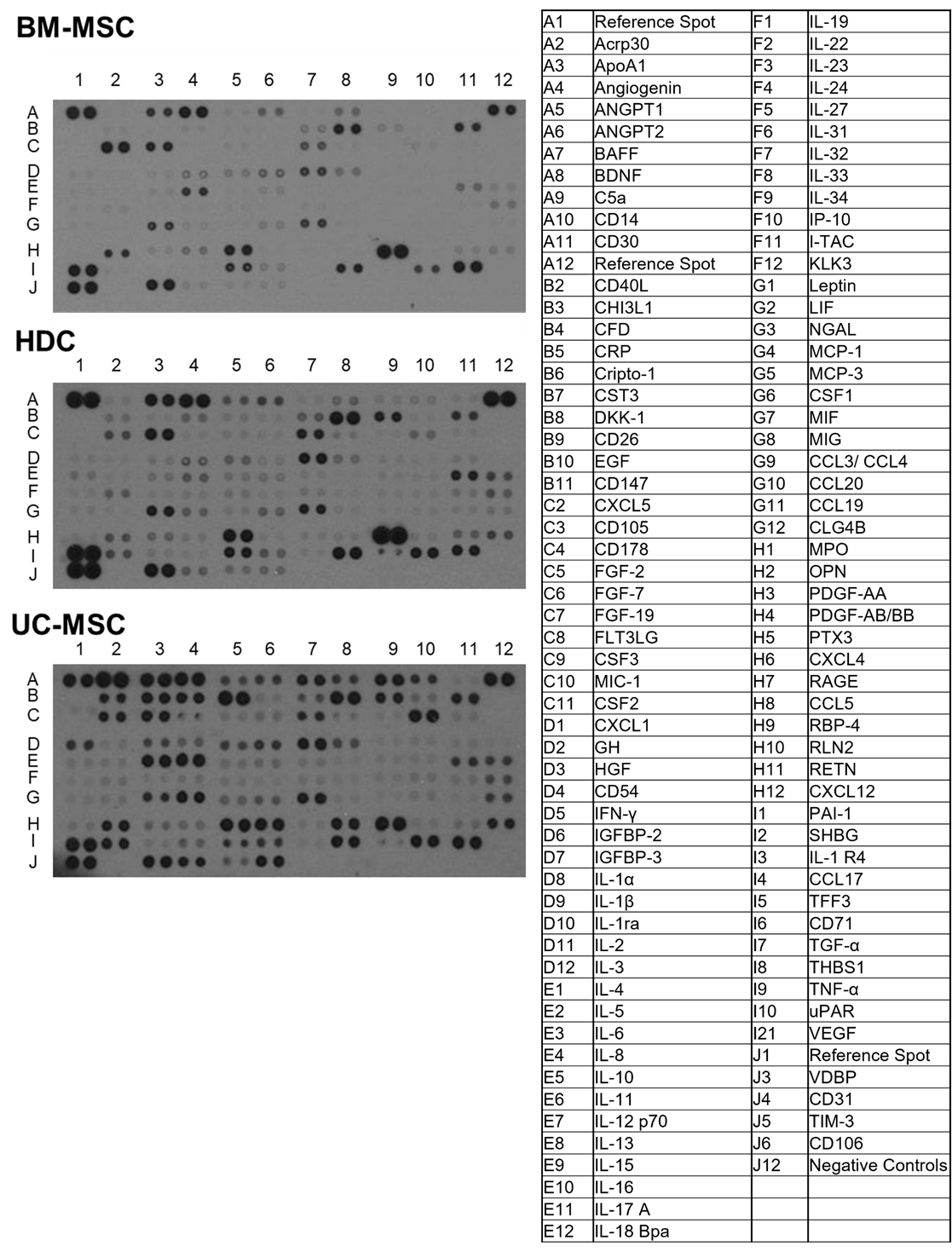


**Figure S1** Cytokine profiling in conditioned media produced by potential COVID-19 cell products. Representative images of the proteome array used to analyze conditioned media produced by BM-MSCs, HDCs, or UC-MSCs. The table on the right panel lists the position coordinates of cytokine antibody on the array.


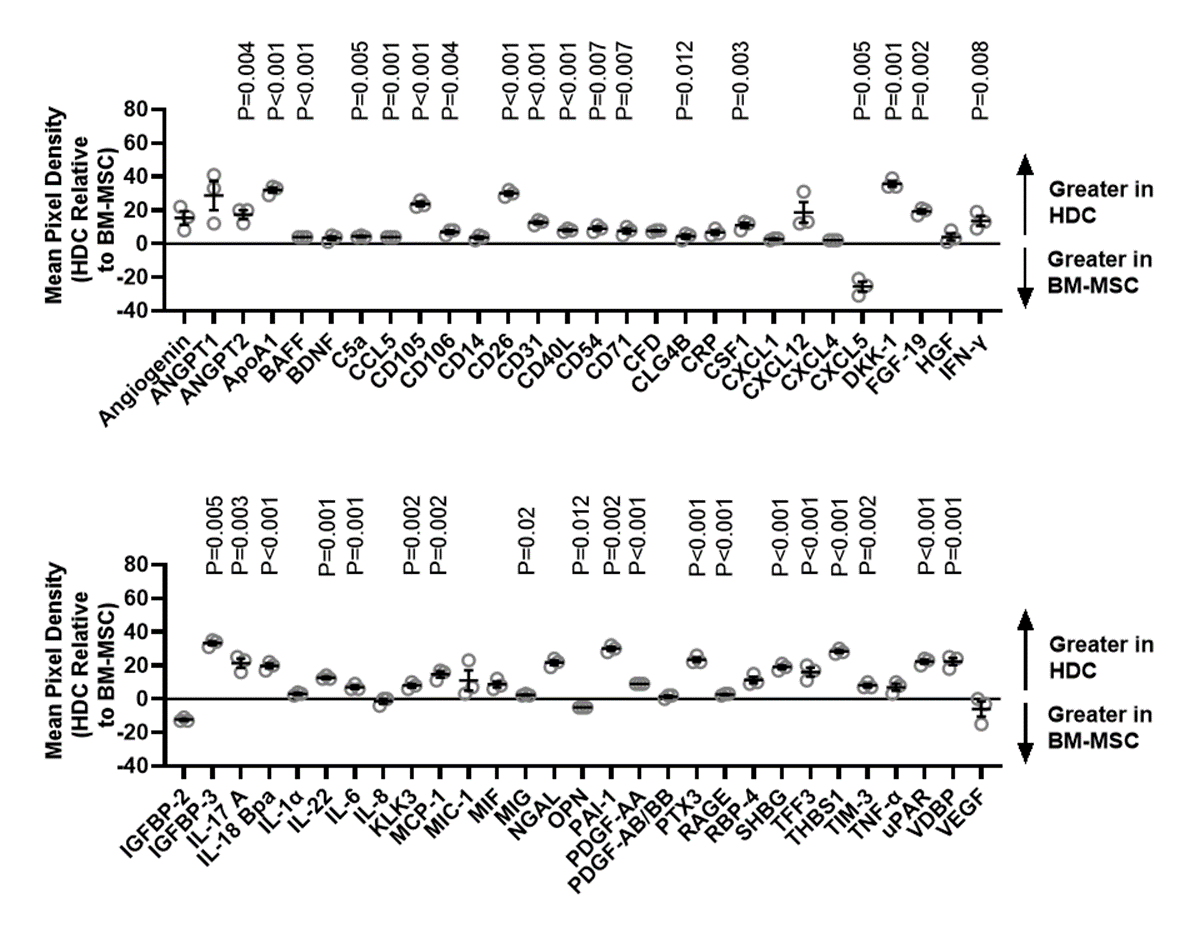


**Figure S2** Relative cytokine content within HDC conditioned media compared to BM-MSC conditioned media. Expression of cytokines within conditioned media produced by HDCs compared to BM-MSCs. Data were compared using an unpaired t-test with individual variances for samples and a two-stage step-up (Benjamini, Krieger, and Yekutieli) false discovery rate to account for multiple comparisons. All data are presented as individual and mean values ± SEM, n=3 biological replicate; each circle represents one data point from one unique biological replicate. Significance is indicated on the panels.


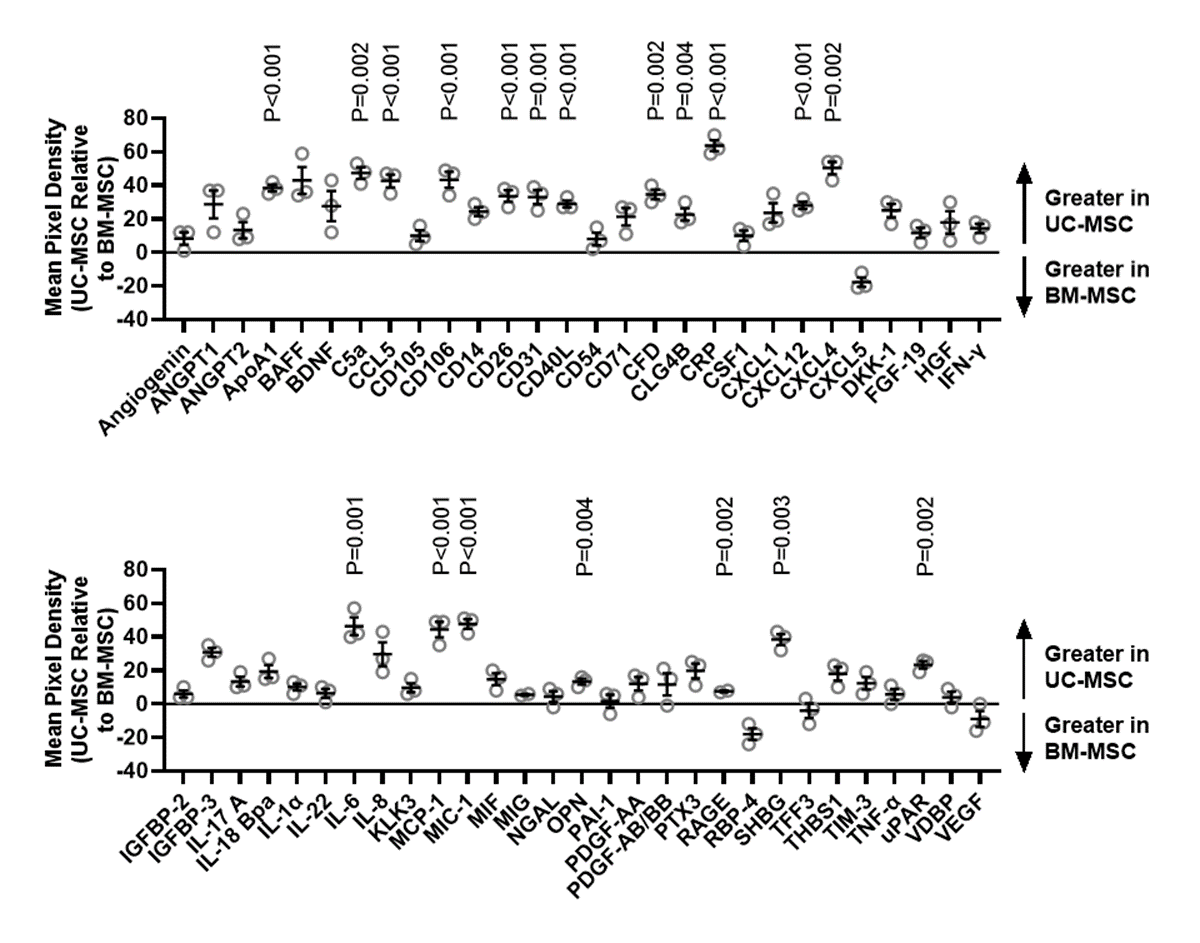


**Figure S3** Relative cytokine content within UC-MSC conditioned media compared to BM-MSC conditioned media. Expression of cytokines within conditioned media produced by UC-MSCs compared to BM-MSCs. Data were compared using an unpaired t-test with individual variances for samples and a two-stage step-up (Benjamini, Krieger, and Yekutieli) false discovery rate to account for multiple comparisons. All data are presented as individual and mean values ± SEM, n=3 biological replicates; each circle represents one data point from one unique biological replicate. Significance is indicated on the panels.


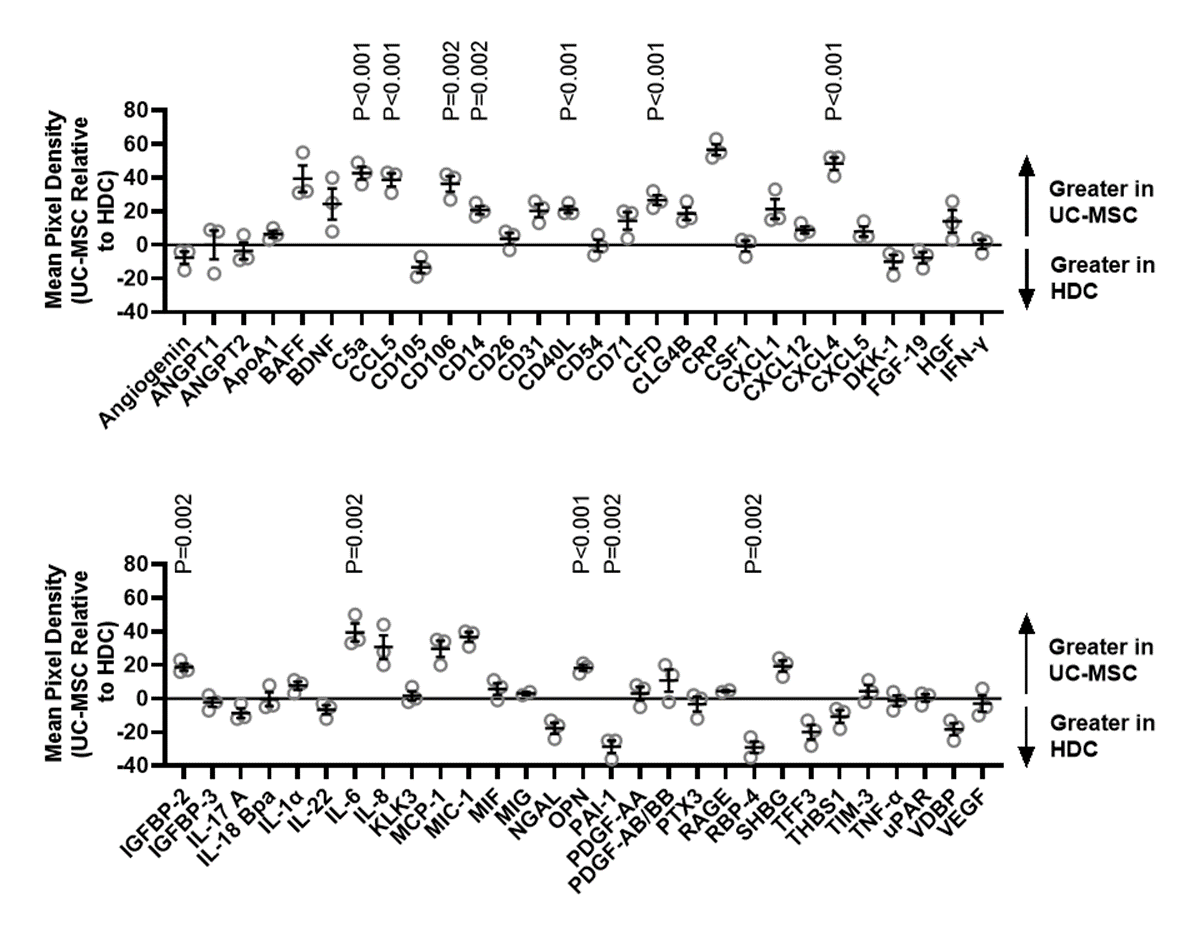


**Figure S4** Relative cytokine content within UC-MSC conditioned media compared to HDC conditioned media. Expression of cytokines within conditioned media produced by UC-MSCs compared to HDCs. Data were compared using an unpaired t-test with individual variances for samples and a two-stage step-up (Benjamini, Krieger, and Yekutieli) false discovery rate to account for multiple comparisons. All data are presented as individual and mean values ± SEM with n=3 biological replicates; each circle represents one data point from one unique biological replicate. Significance is indicated on the panels.


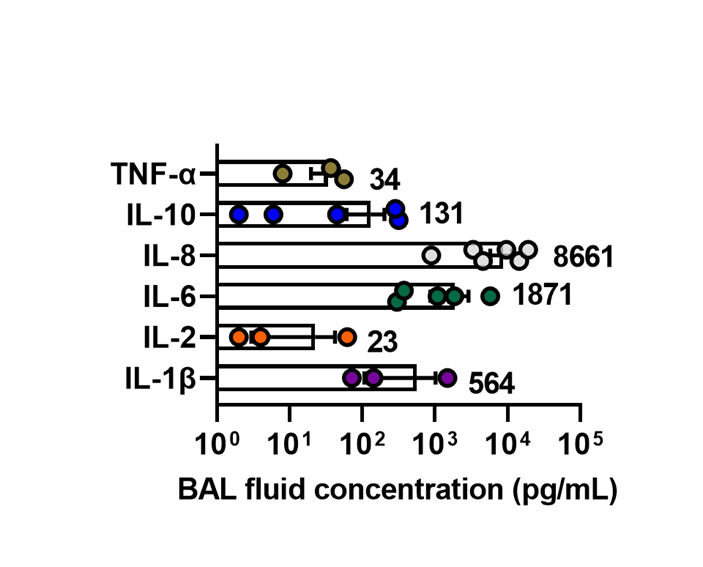


**Figure S5** Cytokine concentrations found within the lung of critically ill COVID-19 patients. This figure depicts literature search results of cytokine concentrations found in bronchial alveolar lavage fluid from critically ill patients with COVID-19. When data were not presented in a tabular form, they were extracted from the figures by using online tool WebPlot Digitizer. All data are presented as individual and mean values ± SEM. The values on the bar graphs represent arithmetic mean of all studies for respective cytokine.


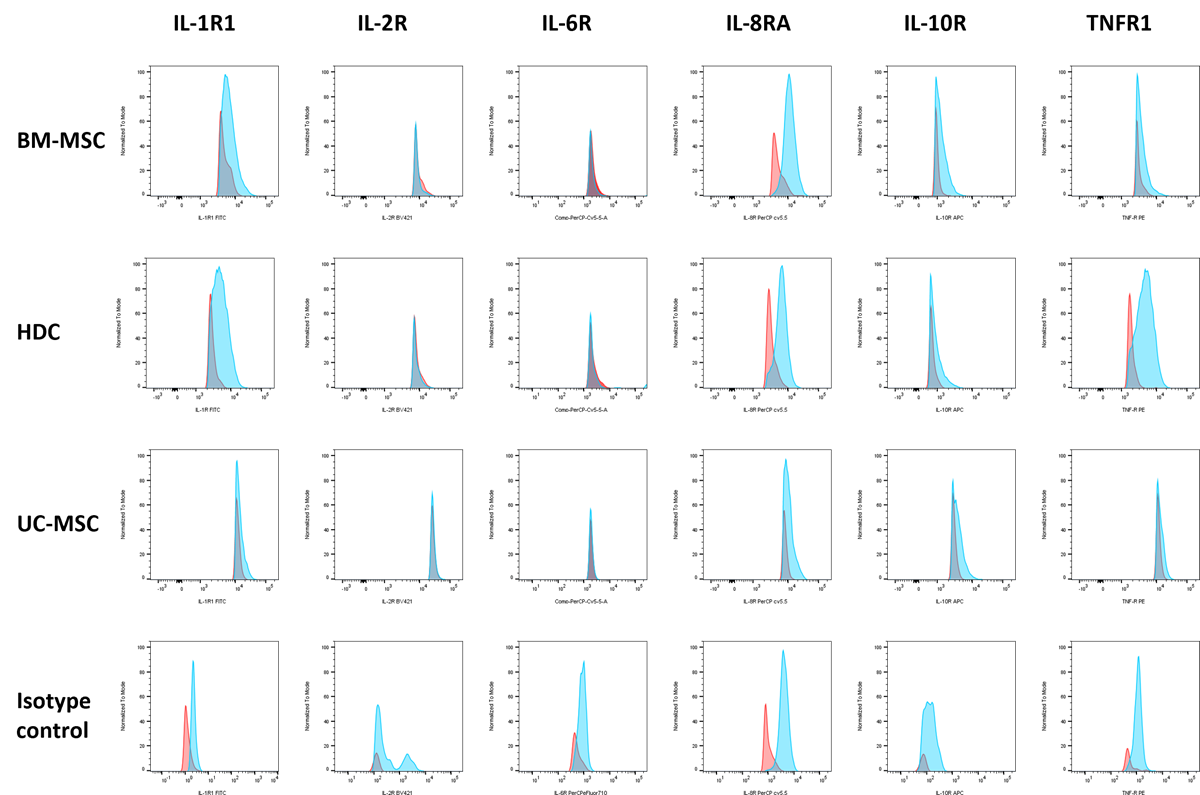


**Figure S6** Flow cytometry showing co-segregation of receptors for COVID-19 related cytokines. Representative flow cytometry images demonstrating detection of FITC anti-human IL-1R1, BV421 anti-human IL-2Rβ, PerCP-eFluor710 anti-human IL-6R, PerCP-cy5.5 anti-human IL-8RA, APC anti-human IL-10R, and PE anti-human TNFR1on BM-MSCs, HDCs and UC-MSCs. Isotype controls were used to correct compensation and confirm antibody specificity.


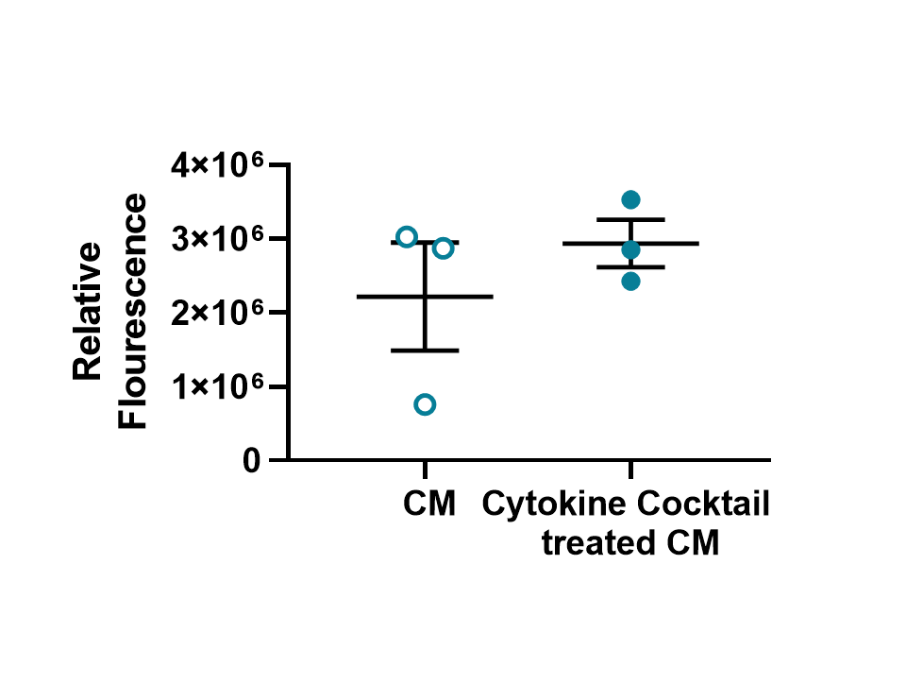


**Figure S7** COVID-19 ARDS cytokine challenged UC-MSC conditioned media effect on pulmonary microvascular endothelial cell permeability. Endothelial cell permeability assay showed that treating pulmonary microvascular endothelial cells with conditioned media (CM) collected from COVID-19 ARDS cytokine cocktail challenged UC-MSCs does not alter endothelial cell permeability. Filled circles represent cytokine cocktail treatment, Hollow circles represent no cytokine cocktail treatment. All data are presented as individual and mean values ± SEM. n=3 biological replicates; each circle represents one data point from one unique biological replicate.
